# Characterization of Chemical Compounds with Antioxidant and Cytotoxic Activities in *Bougainvillea x buttiana* Holttum and Standl, (var. Rose) Extracts

**DOI:** 10.3390/antiox5040045

**Published:** 2016-12-02

**Authors:** Rodolfo Abarca-Vargas, Carlos F. Peña Malacara, Vera L. Petricevich

**Affiliations:** 1 Facultad de Medicina, Universidad Autónoma del Estado de Morelos (UAEM), Calle Leñeros, Esquina Iztaccíhuatl s/n, Cuernavaca, Morelos, C.P. 62350, Mexico; rodolfo.abarca@uaem.mx; 2 Departamento de Ingeniería Celular y Biocatálisis, Instituto de Biotecnología, Universidad Nacional Autónoma de México, Cuernavaca, Morelos, C.P. 62250, Mexico; carlosf@ibt.unam.mx

**Keywords:** solvent extraction, total phenolic content (TPC), antioxidant, cytotoxic, *Bougainvillea x buttiana*

## Abstract

*Bougainvillea* is widely used in traditional Mexican medicine to treat several diseases. This study was designed to characterize the chemical constituents of *B. x buttiana* extracts with antioxidant and cytotoxic activities using different solvents. The extraction solvents used were as follows: distilled water (dH_2_O), methanol (MeOH), acetone (DMK), ethanol (EtOH), ethyl acetate (EtOAc), dichloromethane (DCM), and hexane (Hex) (100%) at an extraction temperature of 26 °C. Analysis of bioactive compounds present in the *B. x buttiana* extracts included the application of common phytochemical screening assays, GC-MS analysis, and cytotoxicity and antioxidant assays. The results show that the highest extraction yield was observed with water and methanol. The maximum total phenolic content amount and highest antioxidant potential were obtained when extraction with methanol was used. With the exceptions of water and ethanol extractions, all other extracts showed cytotoxicity ranging between 31% and 50%. The prevailing compounds in water, methanol, ethanol, and acetone solvents were as follows: 4H-pyran-4-one, 2,3-dihydro-3, 5-dihydroxy-6-methyl (2), 2-propenoic acid, 3-(2-hydrophenyl)-(E)- (3), and 3-*O*-methyl-d-glucose (6). By contrast, the major components in the experiments using solvents such as EtOH, DMK, EtOAc, DCM, and Hex were n-hexadecanoic acid (8), 9,12-octadecadienoic acid (Z,Z) (12); 9-octadecenoic acid (E)- (13), and stigmasta-5,22-dien-3-ol (28).

## 1. Introduction

According to the World Health Organization (WHO), more than 80% of the world’s population relies on traditional medicine for their primary healthcare needs. *Bougainvillea* is a native plant of South America, a type of bush belonging to the Nyctaginaceae family. It is a predominant species in the vegetation of Temixco, Morelos, and other states of México. *Bougainvillea x buttiana* and *B. spectabilis* are widely used in traditional Mexican medicine through preparations, decoctions, infusions, and powders for the treatment of several diseases, including painful disorders, such as cough, bronchitis, respiratory infections, hyperacidity gastritis, peptic ulcers, stomach cramps, fever, diarrhea, injuries, diabetes, and stomachache. *B. x buttiana* extracts are a complex mixture of a large number of phytochemicals that include phenolic compounds and flavonoids [1,2]. It has been reported that the bracts of different colors from *B. x buttiana* present various pharmacological effects, such as antinociceptive, anti-inflammatory, and antioxidant activities, which can be attributed to the presence of phenolic compounds and flavonoids in its extracts [1,2].

Many researchers have reported the influence of different extraction solvents and techniques on the content of natural antioxidants in extracts. Phytochemical compounds, such as flavonoids, phenols, saponins, and tannins, are considered major secondary metabolites in plants. Phenolic compounds, which have a broad spectrum of biochemical activities, represent the largest group [1,2,3,4,5,6,7].

Interest in these classes of compounds are due to their pharmacological activities as radical scavengers [8]. Recently, different studies have shown that phenols and non-phenolic compounds are of great interest to the pharmaceutical industry for their anti-inflammatory, anti-aging, and antimicrobial benefits, which make them an important source of molecules for new drug discovery [9]. Phytochemical studies on plant materials are required to optimize the concentration of known constituents [10]. One important step for utilizing bioactive compounds from plant resources is the extraction process. Selection of the extraction process is an important step for the standardization of herbal products, as they can be utilized in the removal of desirable soluble constituents. Various parameters during the extraction process are critical, including maceration, percolation, infusion, decoction, and so on [11]. Due to the wide variations in the structures and polarities of chemical compounds, extraction from plant products is complex and challenging. The critical extraction parameters include solvent, time, solid-to-solvent ratio, number of extractions, temperature, and partial size of the sample material [9]. Selection of the extraction solvent depends on the specific nature of the bioactive compound being targeted. The extraction yield and, consequently, the biological activity of vegetal extracts can be strongly affected by the solvent applied [12]. For bioactive compound extraction, different solvents, including organic and/or aqueous solutions, have been reported [13]. Recent studies by Quezada and Cherian in 2012 [14] showed that the amount of total phenolic content extracted from flaxseeds was affected by the solvent polarity; for example, solvents with low polarity, such as ethyl acetate, are less efficient than more polar solvents. Water and ethanol are often recommended for extract preparation because of their differences in polarity. The use of organic solvents for industrial extractions has several disadvantages, such as: (a) solvent residue in the product; (b) worker exposure; (c) disposal of waste solvents; and (d) environmental pollution [15]. Several studies have shown that ethanol and boiling water are effective for polyphenol extraction [16,17]. Other studies have shown that water alone can dissolve polysaccharides at a high pressure and temperature. Furthermore, the highest concentrations of ethanol may be limited by temperature.

In this context, the aim of the present study was to identify chemical compounds with antioxidant and cytotoxicity activities in *B. x buttiana* using different extraction solvents.

## 2. Materials and Methods

### 2.1. Chemicals

Methanol, acetone, ethyl acetate, hexane, and dichloromethane were purchased from JT Baker (Philipsburg, NJ, USA). Absolute ethanol was obtained from Golden Bell (Zapopan, Jalisco, Mexico). Gallic acid, Ciocalteu’s Folin Phenol Reagent, Tween-20 and sodium carbonate were purchased from Sigma-Aldrich (Toluca, Mexico). RPMI-1640 medium was obtained from Lonza (Walkersville, MD, USA). Fetal calf serum was obtained from Gibco Life Technologies Corporation (Grand Island, NY, USA). L-929 cells were obtained from American Type Culture Collection (ATCC) (Manassas, VA, USA).

### 2.2. Plant Material

Air parts (flowers) of plants were collected from Temixco, Morelos, (18°52′20.1″ N and 99°14′40.6″ W, 1185 m) in July 2015 and identified as *Bougainvillea x buttiana* Holttum and Standl, (var. Rose). A voucher specimen (33872) was deposited at the Herbarium HUMO, CIByC (UAEM, Cuernavaca, Morelos, México).

### 2.3. Extraction, Preparation, Extraction Solvent, Temperature, and Cycles

*B. x buttiana* (var. Rose) (BxbVR; 500 g) bracts were collected and air-dried at room temperature for one week and immediately subjected to an electric grinder to obtain a fine powder.

For different extractions, 5 g of powered BxbVR was individually mixed with 100 mL of distilled water, methanol, acetone, ethanol, ethyl acetate, dichloromethane, or hexane at 26 °C for 24 h. Liquid extracts obtained were separated from the solid residue at 60 °C using a rotary evaporator (Heidolph, Elk Grove Village, IL, USA) under reduced pressure. All preparations were performed in triplicate. Extracts from each extraction process were collected in separate extraction vials and maintained at room temperature until subjected to phytochemical assays.

Dried extracts were weighed to calculate the percent yield of crude extract by the following Equation (1):

(1) $$\%\;\mathrm{yield}=\frac{A}{B}\times 100$$

where *A* = the total weight of dried extracts obtained after drying and *B* = the total weight of ground plant material taken for each extraction process.

### 2.4. Phytochemical Screening

#### 2.4.1. Total Phenolic Content (TPC)

The total phenolic content present in *B. x buttiana* (var. Rose) extracts was determined using the Folin-Ciocalteau phenol reagent method, as described by Singleton et al. [18]. In brief, 100 μL of extract (100 μg) was added to 0.5 mL of Folin-Ciocalteau phenol reagent and 1 mL of sodium carbonate (7.5% *w*/*v*) to a final volume of 2 mL. The contents were mixed and allowed to stand for 30 min. The absorbance at 765 nm was determined. The TPC is expressed in terms of gallic acid (GA) (equivalents of GA/gram of extract). The following equation is based on the calibration curve: *Y* = 7.5397*X* − 0.0291, *R*^2^ = 0.9993, where *X* is the absorbance and *Y* is the mgGA/g. Each sample was analyzed in quadruplicate.

#### 2.4.2. GC-MS Analysis

Analyses were carried out on an Agilent 6890B gas chromatograph (Hewlett-Packard, Palo Alto, CA, USA) fitted with an HP-5MS fused silica column (5% phenyl methyl polysiloxane 30 m × 0.25 mm i.d., film thickness 0.25 μm), interfaced with an Agilent mass selective detector 5973N and operated by HP Enhanced ChemStation software (Hewlett-Packard, Palo Alto, CA, USA). For GC-MS detection, an electron ionization system with an ionization energy of 70 eV was used. Helium was used as the carrier gas at a flow rate of 1 mL/min. The injector and transfer line temperatures were set at 250 °C and 285 °C, respectively. The column temperature was initially kept at 10 °C for 1 min, then gradually increased to 250 °C at a rate of 5 °C/min, and finally raised to 285 °C at a rate of 1 °C/min. A diluted sample in dimethyl sulfoxide (DMSO) (20% solution) of 2.0 μL was injected into the split mode at a ratio of 1:50. The chromatographic conditions and column used for GC analyses (Agilent 5973N gas chromatograph with FID detector) were the same as those for GC-MS analyses. The identity of the components of the essential oil was assigned by comparison on the HP-5MS capillary column and the GC-MS spectra from the Wiely7Nist data by co-injection with authentic compounds (Sigma Aldrich, Toluca, México). Quantification of the components was performed on the basis their GC peak areas on the HP-5MS column.

### 2.5. Evaluation of Cytotoxicity

L-929 cells obtained from (ATCC) are maintained in T-12.5 cm^2^ filter cap flasks in RPMI-1640 medium supplemented with 1% penicillin streptomycin/l-glutamine and 10% fetal calf serum (FCS) and 5% CO_2_. A monolayer of L-929 cells in medium RPMI-1640 supplemented with 5% of FCS were trypsinized, and distributed with 3−5 × 10^4^ cells per well, when the cells had reached about 90% of confluence, for control cells 100 µL of medium and for samples with 100 µg/100 µL of different extracts diluted in RPMI-1640 medium was added to each well. After incubation for 24, 48, and 72 h at 37 °C in a humidified atmosphere of 5% CO_2_, the supernatants were removed and the remaining living cells were assessed after fixing and staining with crystal violet (0.2% in 20% methanol) for 10 min at room temperature. The absorbance at 620 nm of each well was read in a microplate reader. To calculate the cytotoxicity percentage, the following Equation (2) was used [19]:

(2) $${\%\;\mathrm{cytotoxicity}=(A}_{\mathrm{control}}-\frac{A_{\mathrm{sample}}}{A_{\mathrm{control}}})\times 100$$

### 2.6. DPPH Free Radical Scavenging Activity

The antioxidant activities of the *Bxb* extracts and standard were assessed on the basis of the radical scavenging effect of the stable 2,2′-diphenyl-1-picrylhydrazyl (DPPH)-free radical activity using a modified method [20]. The working solutions of the test extracts were prepared from the serial concentrations of 15.6–250 µg/mL and each extract was added at equal volume to methanolic solution of DPPH (0.1 mM). Quercetin was used as a standard in 37.5–150 μg/mL solution and standard solution separately. These solution mixtures were kept in the dark for 30 min, and the optical density was measured at 517 nm using a spectrophotometer. Methanol (1 mL) containing DPPH (0.1 mM) solution was used as a blank. The absorbance was recorded, and the percent inhibition was calculated using the following Equation (3):

(3) $$\%\mathrm{inhibition}\;\mathrm{of}\;\mathrm{DPPH}\;\mathrm{activity}\;=100-\left[{Abs}_{\mathrm{sample}}\;-\frac{{Abs}_{\mathrm{blank}}}{{Abs}_{\mathrm{control}}}\right]\times 100$$

where *Abs*_control_ is the absorbance of the DPPH plus methanol and *Abs*_sample_ is the absorbance of the DPPH plus sample extract/standard.

### 2.7. Statistics

Statistical analyses were carried out using ANOVA, with the level of significance set at *p* < 0.05. Differences among the means were evaluated using a statistical program GraphPad Prism 6 (La Jolla, CA, USA). All experiments were carried out in quadruplicate and repeated at least twice (mean ± standard deviation (SD).

## 3. Results

Extraction is the first step in the analysis of pharmacologically active compounds from plant materials [21]. The polarity of the solvent used for extraction and the method of extraction play vital roles in both the efficiency and efficacy of pharmacological activities [22,23,24]. With the key principle of solubility, phytoconstituents of varying polarities present in plants can be extracted by using appropriate solvents. Taking into account the fundamental principle of solubility, phytoconstituents of varying polarities in plants can be extracted by using appropriate solvents [25]. Other solvents that have been used for medicinal purposes are pure ethanol or a mixture of ethanol and water [26]. The extraction of bioactive compounds from plants has been widely investigated using conventional solvent extraction methods. This study aimed to determine the effect of extraction conditions on the concentration of the total phenolic content (TPC) and the antioxidant and cytotoxic activities of compounds present in *B. x buttiana*.

### 3.1. Effect of Solvent on Extraction Yield

In the present study, the effect of the extraction process on the yield and phytochemical screening of *Bougainvillea x buttiana* var. Rose was evaluated with seven solvents of differing polarities: water, methanol, ethanol, acetone, ethyl acetate, dichloromethane, and hexane (Table 1).

**Figure 1:**
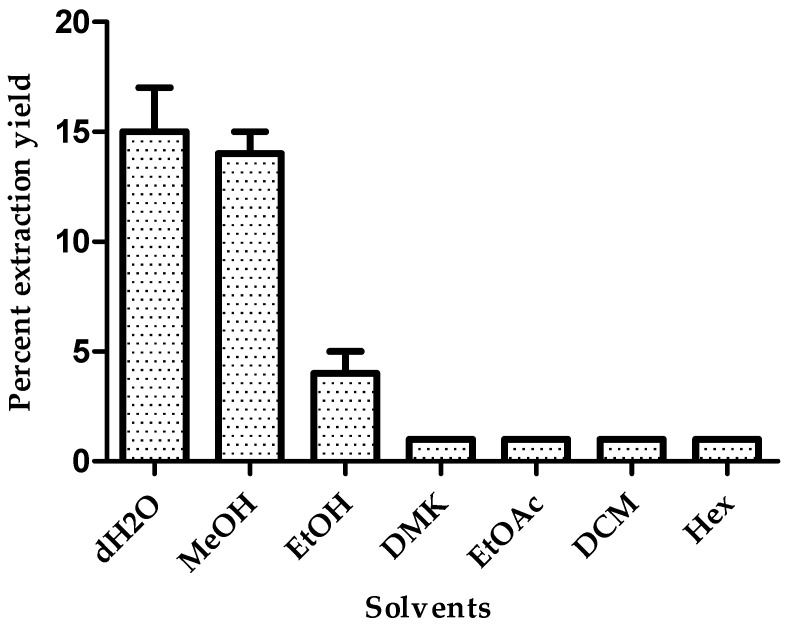
Percent extraction yield. Preparations of *B. x buttiana* with seven different extraction solvents at 100% at 26 °C for 24 h were prepared as described in the Materials and Methods section. Each bar corresponds to the results (mean ± standard deviation (SD)) from three different preparations.

Figure 1 shows the percent extraction yield in each of the solvents up to 100% at 26 °C for 24 h. The values from the three samples of *B. x buttiana* were analyzed in triplicate, and the processes used different solvents at 100% with decreasing polarities. The BxbVR extraction yield increased with the increase in polarity of solvent used. The maximum extraction yield was observed using distilled water and methanol. The ranking order for the percent extraction yield was: dH_2_O = MeOH > EtOH > DCM = DMK = EtOAC = Hex. These results suggest that the major phytochemicals in BxbVR are mostly high in polarity and soluble in H_2_O and methanol. Solvent selection is based on polarity. In this study, different colors of extract were observed. The color of the water extract was dark-brownish; that of methanol was light-brownish; and that of ethanol was yellowish-green. One possible explanation for this observation is the presence of betacyanins, which can easily be isolated from *Bougainvillea* bract plants [27,28].

### 3.2. Effect of Solvent Extraction on TPC Levels

Several investigations have reported that different solvent combinations can be used to extract antioxidants from plant materials. For the extraction of phenolic compounds, the solvents most widely used are water, ethanol, methanol, acetone, and their water mixtures [29,30]. The chemical nature of the phenolic compounds, the extraction method employed, the sample particle size, the storage time and conditions, as well as the presence of interfering substances can all affect the efficiency of the extraction method [30].

**Figure 2:**
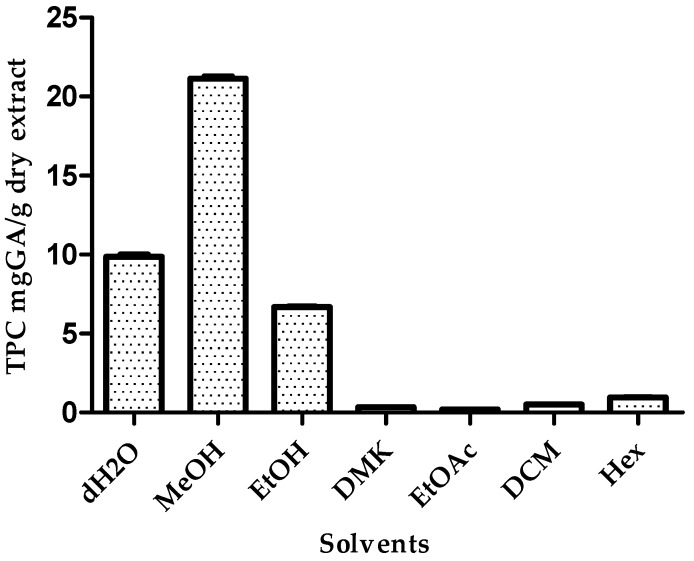
Effect of extraction solvents on the total phenolic content (TPC) amount. Samples of *B. x buttiana* extracts with solvents at 100% at 26 °C for 24 h were assayed to determine the total phenolic content, as described in the Materials and Methods section. Each bar corresponds to the results (mean ± SD) from three different preparations.

The total phenolic content from each *B. x buttiana* extract with solvents at 100% is presented in Figure 2. The highest amount of TPC was obtained by extraction with methanol. The TPC ranged from 0.19 to 21.14 mgGA/g of dry extract. The results obtained from were in the order: MeOH > dH_2_O > EtOH > Hex > DCM = DMK = EtOAc. The TPC is often extracted at higher amounts in more polar solvents. In this study, among the pure solvents, methanol proved to be the most efficient solvent for the isolation of TPC from *B. x buttiana*. Distilled water (PI = 9) is an efficient solvent for the extraction of TPC from the *B. x buttiana* bracts studied. The results have shown that solvents with a similar polarity to ethanol (PI = 5.2) and acetone (PI = 5.1) have significantly different effects on TPC. These significant variations indicate that the changes in phenolic solubility may be altered, which depends on the particular extraction conditions used.

However, the amount of TPC was higher in polar solvents and weaker in non-polar solvents. The variations in TPC obtained from various extracts are attributed to the polarities of different compounds present in the plant materials. In general, the extractability of a particular compound is a function of the ratio of solute to solvent. In this case, the recovery of TPC appeared to be dependent on the type of solvent used, its polarity index, and the solubility of TPC in the extraction solvent. The solubility of polyphenols depends on the hydroxyl groups, molecular size, and length of the hydrocarbon [31].

### 3.3. Effect of Solvent Extraction on Cellular Cytotoxicity

**Figure 3:**
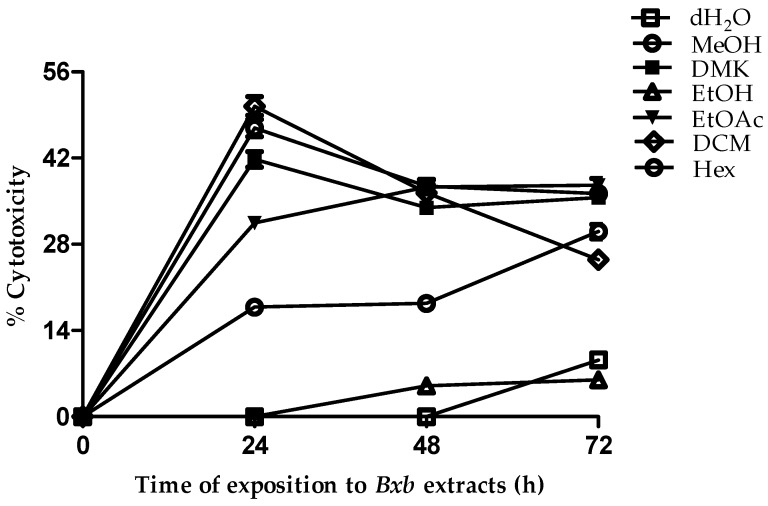
Cytotoxicity. *B. x buttiana* extracts with solvents at 100% at 26 °C for 24, 48 and 72 h were assayed to determine their effects on cellular cytotoxicity, as described in the Materials and Methods section. Each point corresponds to the results (mean ± SD) from three different preparations.

In the present study, cellular cytotoxicity was measured in the presence of 100 µg/100 µL of each solvent extraction. The effects of all extracts with solvents at 100% were evaluated in terms of their cytotoxicity in L-929 cells for different time intervals. The cytotoxicity percentage is presented in Figure 3. At 24 h, the cytotoxicity ranged from 0% to 50.4%, and the ranking order was as follows: DCM > MeOH > DMK > EtOAc > Hex > dH_2_O > EtOH. Increasing the incubation time did not affect cytotoxicity. By contrast, extraction with ethanol yielded 5% and 6% cytotoxicity after 48 h and 72 h, respectively. Several biologically active compounds have been extracted from plant materials using organic solvents, such as hexane, ether, acetonitrile, benzene, and ethanol, at various dilutions [32,33]. However, these solvents may exert toxic effects in human patients, thus limiting their therapeutic potential [34,35].

### 3.4. Effect of Solvent Extraction on Antioxidant Activity

The antioxidant potential is inversely proportional to the IC_50_ value, which is calculated from the linear regression of the antioxidant activity versus the extract concentration. Quercetin, which was used as a standard, showed 50% inhibition at 96.82 μg/mL. The capacity to neutralize 50% of free radicals for BxbVR extraction at 26 °C ranged from 223 to 1455.6 μg/mL, and the antioxidant potential ranking order was as follows: MeOH > EtOH > dH_2_O > DMK > EtOAc > Hex > DCM (Table 2).

### 3.5. GC-MS Analysis

Although the benefits and biological activities of *B. x buttiana* have been reported, little is known scientifically about its antioxidant properties. Exogenous antioxidants from natural sources can improve the function of the endogenous antioxidant system, which is responsible for preventing free radicals in the body [31]. Recently, we reported that extracts contain polyphenols and exert DPPH radical scavenging activity. In the present study, we found that BxbVR extracts using different solvents showed the presence of various components.

To determine the prevalent component present in extracts from different solvents, samples were subjected to GM-chromatography. GC-MS analysis revealed four components present in water extracts, six components in MeOH extracts, nine components in EtOH extracts, eight components in DMK extracts, sixteen components in EtOAc extracts, thirteen components in DCM extracts, and six components in Hex extracts.

The prevalent components, along with important parameters in GC qualitative analysis which are their retention times (RT) and concentrations (%) in BxbVR extracts using different solvents, are presented in Table 3.

The polarity of a molecule is a characteristic value known as the electric moment. Polar substances dissolve in polar substances because it is thermodynamically favorable for the solvent-solute forces. The solubility of a drug in a given solvent is mainly a function of the polarity of the solvent. Solvents may be divided into polar, semi-polar, and non-polar types. Polar solvents dissolve ionic and other polar solutes. The number of compounds characterized by GC-MS is obtained from each solvent extraction. Three compounds were found in dH_2_O (2.57%), MeOH (2.75%), and EtOH (2.11%) extracts. Six polar solutes were found in dH_2_O (96.01%), MeOH (94.17%), EtOH (51.43%), and DMK (16.84%) extracts. Semi-polar solvents, such as alcohols and ketones, may induce a certain degree of polarity in non-polar molecules and may, thus, act to improve the miscibility of polar and non-polar liquids, whereas non-polar solvents dissolve non-polar molecules. Eight compounds were found in EtOH (10.76%), DMK (23.14%), DCM (4.14%), and Hex (26.50%) extracts. Twelve compounds were found in EtOH (6.35%), DMK (19.92%), and DCM (3.37%) extracts.

**Figure 4:**
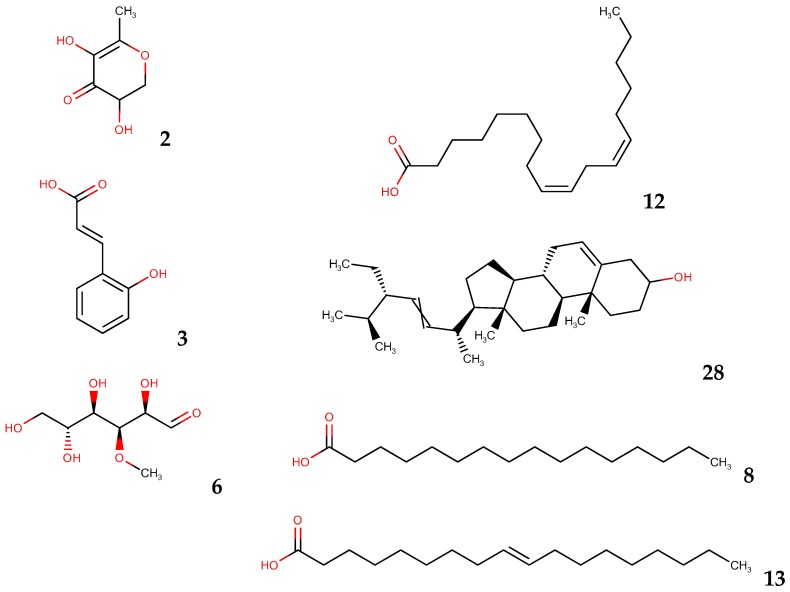
Structures of the compounds.

Figure 4 shows the molecular structures of the major compounds present in *B. x buttiana* extracts. Marvin was used to draw, display, and characterize chemical structures [36].

## 4. Conclusions

The results obtained demonstrate that the high polarity index of 100% methanol is able to extract phenolic compounds with low molecular weights. Additionally, this solvent is effective at extracting compounds with higher antioxidant potential. With the exceptions of water and ethanol, all of the other solvent extractions were cytotoxic (between 31% and 50%). Semi-polar solvents, such as alcohols and ketones, are able for to extract compounds with a degree of polarity. Extraction with hexane, which has a zero index of polarity, was only able to extract lipophilic compounds. The present results of this study do not reveal which chemical compound is responsible for antioxidant activity. Hence, the whole plant could be of use as a good source of antioxidants. Further studies are needed for the isolation and identification of individual compounds from the crude extracts of *Bougainvillea x buttiana* and in vivo studies are needed for better understanding of their mechanism of action as antioxidants.

## Figures and Tables

**Table 1:** Polarity index (Snyder [27]).

Solvent	Polarity Index (PI)
Water (dH_2_O)	9.0
Methanol (MeOH)	6.6
Ethanol (EtOH)	5.2
Acetone (DMK)	5.1
Ethyl acetate (EtOAc)	4.4
Dichloromethane (DCM)	3.7
Hexane (Hex)	0

**Table 2:** IC_50_ μg/mL values.

Solvents	IC_50_ (µg/mL)
dH_2_O	286.80 ± 0.01
MeOH	223.10 ± 0.02
EtOH	232.10 ± 0.02
DMK	300.90 ± 0.05
EtOAc	353.80 ± 0.01
DCM	1455.68 ± 0.19
Hex	373.70 ± 0.03

**Table 3:** Compounds present in different solvent extracts.

N°	Compounds	RT (min)	Solvent‘s						
			dH_2_O	MeOH	EtOH	DMK	EtOAc	DCM	Hex
			GC Area %						
1	2,5-Dimethyl-4-hydroxy-3(2H)-furanone	8.20	0.87						
2	4H-Pyran-4-one, 2,3-dihydro-3,5-dihydroxy-6-methyl-	9.55	0.48	0.62					
3	2-Propenoic acid, 3-(2-hydrophenyl)-(E)-	10.61	2.57	2.75	2.11				
4	Ethanone, 1-(2-hydroxy-5-methylphenyl)-	12.04		0.51					
5	Phenol, 2,4-bis(1,1-dimethylethyl)-	14.57							9.88
6	3-*O*-Methyl-d-glucose	17.19	96.01	94.17	51.44	16.80			
7	Tetradecanoic acid	17.38					1.92		
8	n-Hexadecanoic acid	19.49			10.76	23.10	12.40	4.14	26.50
9	Hexadecanoic acid, ethyl ester	19.8					1.96		
10	Naphthalene, 3,4-dihydro-1,8-bis (trimethylsilyloxy)-	19.99			8.94				
11	2-(Phenyl-piperidin-1-yl-methyl)-cyclohexanol	21.16		0.86					
12	9,12-Octadecadienoic acid (Z,Z)-	21.16			6.35	19.90	14.05	3.37	
13	9-Octadecenoic acid (E)-	21.21			10.40		10.64		
14	Octadecanoic acid	21.35			1.35			1.32	5.29
15	9,12-Octadecadienoic acid, ethyl ester	21.39			0.90		2.95		
16	9,12,15-Octadecatrienoic acid, ethyl ester, (Z,Z,Z)-	21.46					2.93		
17	Pentacosane	24.88						1.49	
18	Heptacosane	28.16					2.56	2.50	
19	Oxirane, heptadecyl-	30.04						2.93	
20	Hexacosane, 9-octyl-	30.33					2.42	1.86	
21	Nonacosane	30.76					5.33	7.61	
22	1,30-Triacontanediol	32.56					4.45	11.30	14.47
23	1-Dotriacontanol	33.46				19.90	16.07		18.07
24	1-Hexacosene	33.59						29.87	
25	Vitamin E	34.18					2.93		
26	Cyclooctacosane	35.34					4.36		
27	1-Triacontanol	37.34					10.39	22.21	
28	Stigmasta-5,22-dien-3-ol	37.34			7.72	10.60	4.63	2.53	25.81
29	Chondrillasterol	37.55		1.02				8.98	
30	Stigmast-7-en-3-ol, (3β,5α)	38.86				9.62			
